# Investigation of Low-Cost FDM-Printed Polymers for Elevated-Temperature Applications

**DOI:** 10.3390/polym14142826

**Published:** 2022-07-11

**Authors:** Jan Lukas Storck, Guido Ehrmann, Uwe Güth, Jana Uthoff, Sarah Vanessa Homburg, Tomasz Blachowicz, Andrea Ehrmann

**Affiliations:** 1 Faculty of Engineering and Mathematics, Bielefeld University of Applied Sciences, 33619 Bielefeld, Germany; jan_lukas.storck@fh-bielefeld.de (J.L.S.); jana.uthoff@fh-bielefeld.de (J.U.); sarah_vanessa.homburg@fh-bielefeld.de (S.V.H.); 2 Virtual Institute of Applied Research on Advanced Materials (VIARAM); guido.ehrmann@gmx.de; 3 Department of Physical and Biophysical Chemistry (PC III), Faculty of Chemistry, Bielefeld University, 33615 Bielefeld, Germany; uwe.gueth@uni-bielefeld.de; 4 Institute of Physics—Center for Science and Education, Silesian University of Technology, 44-100 Gliwice, Poland; tomasz.blachowicz@polsl.pl

**Keywords:** additive manufacturing, polymers, space, microsatellites, thermal stability, dimensions, mechanical properties

## Abstract

While fused deposition modeling (FDM) and other relatively inexpensive 3D printing methods are nowadays used in many applications, the possible areas of using FDM-printed objects are still limited due to mechanical and thermal constraints. Applications for space, e.g., for microsatellites, are restricted by the usually insufficient heat resistance of the typical FDM printing materials. Printing high-temperature polymers, on the other hand, necessitates special FDM printers, which are not always available. Here, we show investigations of common polymers, processible on low-cost FDM printers, under elevated temperatures of up to 160 °C for single treatments. The polymers with the highest dimensional stability and mechanical properties after different temperature treatments were periodically heat-treated between -40 °C and +80 °C in cycles of 90 min, similar to the temperature cycles a microsatellite in the low Earth orbit (LEO) experiences. While none of the materials under investigation fully maintains its dimensions and mechanical properties, filled poly(lactic acid) (PLA) filaments were found most suitable for applications under these thermal conditions.

## 1. Introduction

Three-dimensional printing belongs to the group of technologies that nowadays affect production and even design processes. The nearly unlimited degrees of freedom, combined with not requiring expensive molds, enable the production of individual and complex shapes [1]. In particular, the fused deposition modeling (FDM) process is often found in research, industry, and even privately due to its ease of use and the inexpensive printers and materials [2]. With FDM, several polymers can be transformed into desired shapes, while other 3D printing techniques enable also the processing of metals, hydrogels, etc. [3,4]. 

Beyond rapid prototyping, typical applications of 3D-printed objects are membrane technology, drug delivery, biotechnology, composites with textiles and other materials, or even space missions [5,6,7,8,9]. Many of these applications require the use of polymers, which show a certain thermal stability, i.e., very low deformation and change in the mechanical properties upon heating. Several research groups thus reported the improved thermal stability of FDM-printed materials, e.g., by blending polymers with graphite nanoplatelets [10], graphene oxide [11], or carbon fibers [12]. One of the typical temperatures necessary for biotechnological applications is 121 °C, used for autoclaving, which often prevents some typical biocompatible low-cost FDM printing materials, such as PLA, from being used in this area [13,14,15]. On the other hand, applications in space necessitate working with materials that are temperature-resistant against heat and also coldness, which may be complicated for polymers and often results in using metal 3D printing for these applications [16,17,18]. However, in particular, microsatellite parts are often prepared in universities and partly even schools, which normally do not have access to metal printing [19]. Such microsatellites have to withstand high and low temperatures, typically in the range of −100 °C to +100 °C for satellites in the low Earth orbit (LEO) [20]. Both metals and polymers are not easily chosen for such conditions—while metals may be impaired by atomic oxygen in the LEO and impaired by cosmic rays, many polymers have glass transition temperatures that are too low or suffer from mechanical problems upon temperature cycling [21,22]. This is why polyether ether ketone (PEEK) is often used, a high-temperature material that was suggested as a heat shield for the re-entry of microsatellites since it could withstand temperature cycles between −70 °C and +140 °C [23]. In another study, further high-temperature polymers were tested, such as polyetherimide (PEI) ULTEM 9085, PEI-modified ULTEM 1010, or poly(ether ketone ketone) (PEKK), and also found suitable for similar thermal conditions [24]. These materials, however, can only be printed in specialized, large, and expensive FDM printers, which are not as abundantly available as common FDM printers for more usual polymers [25]. For the latter, however, only a few reports exist on the mechanical and dimensional stability under thermal cycling or at elevated temperatures in general.

In a previous paper [26], we investigated several materials processible on common low-cost FDM and additionally stereolithography (SLA) printers with respect to their dimensional stability and mechanical properties after slow cycles (average heating/cooling rates of 10 K/h) between −15 °C and +85 °C or +185 °C, respectively. While only ABS and one of the SLA-printed materials could withstand a temperature of 185 °C without a significant decrease in the maximum force in three-point bending tests, high-temperature poly(lactic acid) (HT-PLA) could withstand 85 °C without problems, suggesting more tests with different PLA materials.

Here, we report a more thorough investigation of low-cost 3D printing polymers by extending the previous study to several more FDM-printable materials, investigating their dimensional stability and mechanical properties after slow heating to defined temperatures, as well as during periodic thermal treatment between −40 °C and +80 °C in cycles of 90 min. The obtained results contribute to an improved understanding of the behavior of low-cost FDM polymers at elevated temperatures and can serve as a basis for the selection of materials for, e.g., microsatellites in LEO.

## 2. Materials and Methods

The specimens under investigation were printed on the FDM printers I3 MK3 (Prusa Research A.S., Prague, Czech; all pure PLA samples), CR-10 V2 (Creality, Shenzhen, China; PLA “Coffee” samples), and Orcabot XXL (Prodim, The Netherlands; all other samples). Nozzle diameters were 0.4 mm for all printers; layer heights were chosen as 0.15 mm, if not indicated otherwise. The specimens were printed with two perimeters and a linear infill pattern (100% at an angle of ±45°). The testing length of the 3-point bending test of 80 mm corresponded to sample dimensions of 4 mm × 10 mm × 100 mm according to DIN EN ISO 178. For the test series, the following filaments were chosen and printed with the given nozzle/bed temperatures, which were chosen according to the manufacturers’ specifications and according to experience with the printer filament combinations to ensure high quality of printed specimens:

1. PLA black (Verbatim, Eschborn, Germany), 215 °C/75 °C;
2. PLA red (Fil-A-Gehr, produced by Gehr, Mannheim, Germany), 215 °C/75 °C;
3. PLA grey (Prusa), 215 °C/75 °C;
4. PLA “Coffee” (Proto-Pasta, Vancouver, WA), 205 °C/60 °C;
5. PLA “UV” (esun, Shenzhen, China), 210 °C/60 °C;
6. PLA “temp” (esun), 210 °C/60 °C;
7. HT-PLA (Filamentworld, Neu-Ulm, Germany), 215 °C/60 °C;
8. Acrylonitrile-butadiene-styrene (ABS) (Filamentworld), 230 °C/90 °C;
9. High-impact polystyrene (HIPS) (esun), 250 °C/90 °C;
10. Polyethylene terephthalate glycol (PETG) (Filamentworld), 230 °C/60 °C;
11. PETG “Glow in the dark” (extrudr, Lauterach, Austria), 210 °C/60 °C;
12. HDglass (a special PETG, FormFutura), 215 °C/60 °C;
13. HDglass “Carbon Fil” (FormFutura, Nijmegen, The Netherlands), 230 °C/60 °C.

All samples were subjected to thermal treatment at 100 °C, 120 °C, 140 °C and 160 °C in the prequalification tests (higher temperatures were neglected for materials molten or strongly deformed at lower temperatures). Prequalification tests were performed in a muffle oven B150 (Nabertherm, Lilienthal, Germany) by approaching the mentioned temperatures with rates of 100 K/h, followed by isothermal treatment for 1 h and slowly letting the samples cool down in the closed oven, typically resulting in 8-12 h of cooling. Subsequently, 3-point bending tests were performed on a universal testing machine (Kern & Sohn GmbH, Balingen-Frommern, Germany) with 10 mm/min speed. Dimensional stability was investigated by averaging over three measurements of height, width and length per sample, respectively, with a micrometer caliper.

According to the prequalification tests’ results, some of these materials were chosen for the final test. These materials were investigated by differential scanning calorimetry (DSC 3, Mettler-Toledo, Gießen, Germany) to find their cold crystallization temperatures. Half of the samples were afterwards thermally post-treated at the cold crystallization temperature, while the other half were used as printed. Cold crystallization is a typical post-treatment often used for PLA and other FDM printing materials since the fast cooling of the molten polymer during FDM printing reduces crystallization [26,27,28].

These samples were subjected to thermal cycling between +85 °C and −40 °C (the minimum temperature of the climate chamber CTC256 (Memmert, Schwabach, Germany)) within 90 min cycle duration. The first set of samples experienced 12 cycles, while 100 cycles were applied on the second set of samples. Three-point bending tests were performed as described before. Surface investigations of the materials after final tests were performed by scanning electron microscopy (SEM, FEI XL30 ESEM, Philips, Amsterdam, The Netherlands) at a voltage of 13 kV, after sputtering the samples with palladium. Optical examinations of the specimens were performed by a digital microscope, Camcolms2 (Velleman, Gavere, Belgium). 

All tests (prequalification and final tests) were performed in triplicate.

## 3. Results and Discussion

This section firstly describes the results of the prequalification tests on all materials mentioned in the previous section, followed by the final tests on the chosen optimal materials.

### 3.1. Prequalification Tests with All Materials

**Figure 1:**
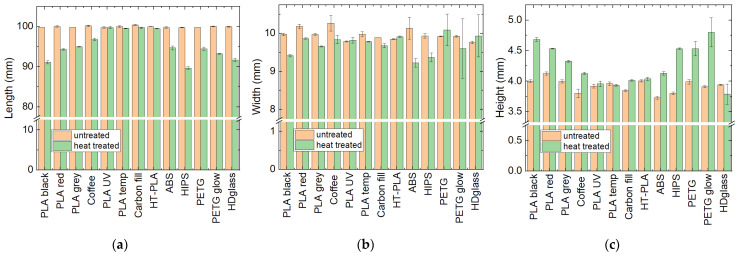
Dimensions of the heat-treated samples: (**a**) length; (**b**) width; (**c**) height. In all graphs, error bars indicate standard deviations.

In the first screening, thermal treatment was performed at temperatures of 100 °C or 140 °C, depending on the results of the previous tests [26] and information from the filament producers about maximum temperatures, i.e., 100 °C for all PLA samples and Carbon Fill and 140 °C for ABS, HIPS and all other PETG samples (including HDglass). Figure 1 shows the results of the dimensional stability tests. Noticeably, all PETG (including HDglass) showed strong deformations in one or more directions due to heating above the glass transition temperature of approximately 75–82 °C [29]. Shrinking was less pronounced for HDglass, while these samples showed strong buckling and a change in the optical properties from translucent to nearly transparent samples, indicating a reduction in the air voids typical for FDM-printed samples. On the other hand, all filled or blended PLA (i.e., PLA “UV”, PLA “temp”, HDglass Carbon Fill and HT-PLA) showed good dimensional stability. Pure PLA, PLA “Coffee”, ABS and HIPS showed clear dimensional variations upon heat treatment. These results suggest the testing of filled PLA materials at higher temperatures and PETG at lower temperatures, while pure PLA, PLA “Coffee”, ABS and HIPS should not be examined further.

**Figure 2:**
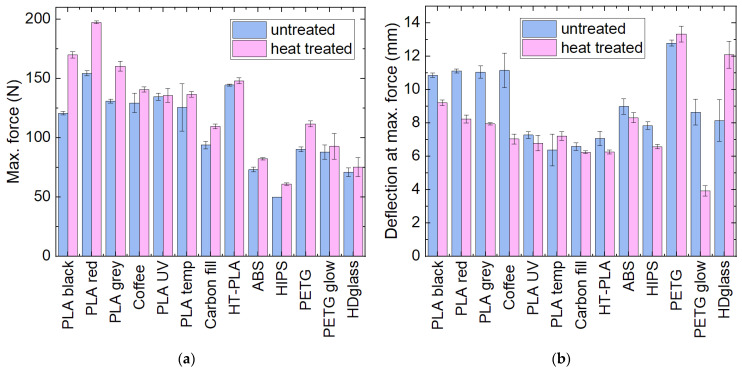
Mechanical properties of the samples as printed and after heat treatment: (**a**) maximum force; (**b**) deflection at maximum force in 3-point bending tests.

Besides the dimensional stability, the mechanical properties have been investigated (Figure 2). Most samples showed an increase in the maximum force combined with a decrease in the deflection at this maximum force upon heat treatment, i.e., they became more brittle, indicating cold crystallization. This is especially valid for the pure PLA samples and PETG “Glow in the dark”. Contrastingly, HDglass showed an increase in deflection at maximum force after heat treatment.

**Figure 3:**
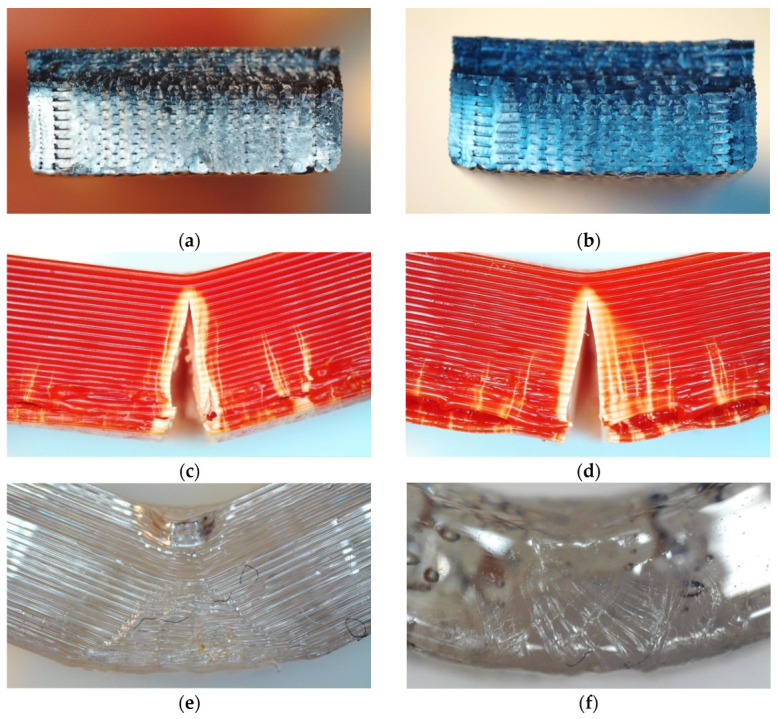
Microscopic images after 3-point bending tests: (**a**) PLA grey as printed; (**b**) PLA grey heat-treated; (**c**) ABS as printed; (**d**) ABS heat-treated; (**e**) HDglass as printed; (**f**) HDglass heat-treated.

To investigate this behavior in more detail, exemplary micrographs after three-point bending tests are depicted in Figure 3. Here, PLA shows stress whitening, followed by brittle failure, in the as-printed samples (Figure 3a) as well as in the heat-treated ones (Figure 3b). ABS, in contrast, shows stress whitening in combination with ductile failure in both cases (Figure 3c,d). Finally, HDglass is strongly modified after heat treatment, as visible by comparing Figure 3e (as-printed sample) with Figure 3f (heat-treated specimen), resulting in the modified mechanical properties, as illustrated in Figure 2b.

These first prequalification tests did not only exclude some materials from further tests, but also posed questions about relaxation processes during heat treatment, which may be responsible for the dimensional changes. Such relaxation can be expected to occur especially in the case of layer heights significantly below the nozzle width, as, in this case, the molten polymer strands are pressed upon each other. A possible test for this is represented by comparing samples printed with different layer heights. Additionally, the heating rate during the thermal treatment may play a certain role, as is known for the thermal stabilization of polymers preceding carbonization [30]. Moreover, HDglass is known to be autoclavable [31] and thus may be interesting for temperatures below the 140 °C tested here; thus it was investigated after heat treatments at 100 °C and 120 °C. Finally, the filled PLA materials were not deformed after heat treatment at 100 °C and thus should also be tested at higher temperatures.

It should be mentioned that while some of these results could be expected from the literature, some others are unexpected. On the one hand, materials such as ABS and the chemically similar HIPS are often regarded as relatively temperature-stable, which could not be verified in our study. In particular, HDglass, often used in applications that necessitate temperatures above 100 °C, showed an unexpectedly strong deformation on the macro- and on the micro-scale, as visible in Figure 3. On the other hand, some of the filled PLA materials performed unexpectedly well, indicating that these materials may be underestimated and could be used for applications necessitating temperatures above approximately 100 °C.

### 3.2. Further Prequalification Tests with Chosen Materials

**Figure 4:**
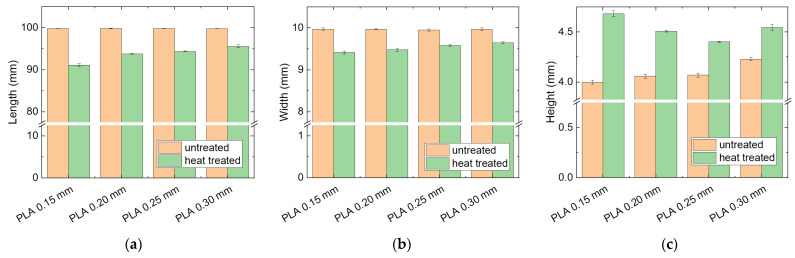
Dimensions of the heat-treated (100 °C) PLA samples with different layer heights: (**a**) length; (**b**) width; (**c**) height.

The results of the dimensional tests on PLA samples with different layer heights are depicted in Figure 4. As estimated, the lengthwise shrinkage and the increase in the height are less pronounced for thicker layers, i.e., these deformations are indeed relaxation effects.

**Figure 5:**
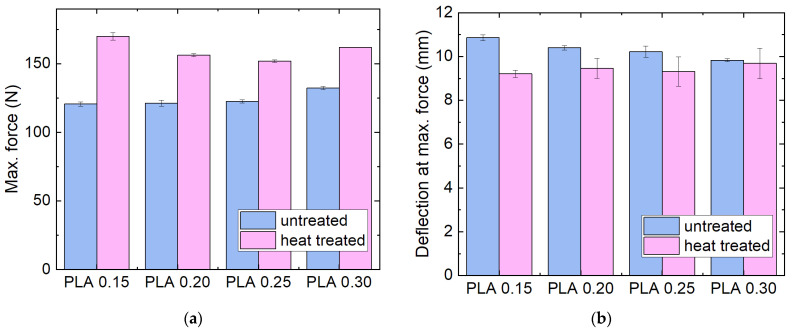
Mechanical properties of the PLA samples with different layer heights, as printed and after heat treatment at 100 °C: (**a**) maximum force; (**b**) deflection at maximum force in 3-point bending tests.

The mechanical properties were also compared for different layer heights, as shown in Figure 5. Here, a similar effect is visible, with a small increase in the maximum force with increasing layer height and a reduction in the positive effect of the heat treatment, i.e., less crystallization upon heat treatment can be expected for thicker layers. The deflection at maximum force shows a similar trend, with the difference between untreated and heat-treated samples becoming smaller for larger layer heights.

**Figure 6:**
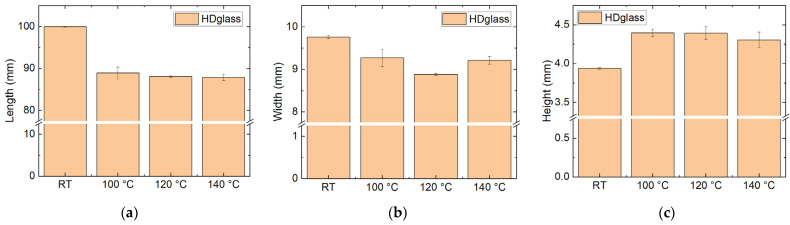
Dimensions of HDglass samples, as printed (“RT”, room temperature) and heat-treated at different temperatures: (**a**) length; (**b**) width; (**c**) height.

HDglass was tested after thermal treatment at different temperatures due to its well-known autoclavability (*T* = 121 °C). The results of the dimensional tests are given in Figure 6. Contrary to expectations for a polymer known for autoclavability, even for heat treatment at only 100 °C, the samples showed strong deformation, i.e., lengthwise shrinkage combined with an increase in height. This finding underlines that this material is not suitable for high-temperature applications in which the dimensional stability is important, as would be the case for applications in space.

**Figure 7:**
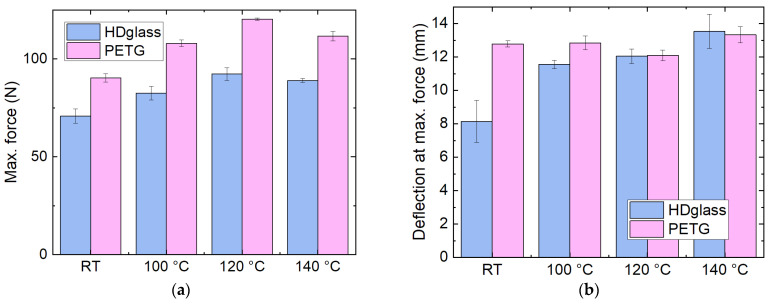
Mechanical properties of HDglass samples, as printed (RT) and heat-treated at different temperatures: (**a**) maximum force; (**b**) deflection at maximum force in 3-point bending tests.

Nevertheless, the mechanical properties of HDglass—and, interestingly, also of PETG, which completely lost its dimensions after heat treatment even at 100 °C—were slightly increased after thermal treatment, as Figure 7 shows. These results indicate the expected crystallization due to heat treatment. Nevertheless, PETG and HDglass were not further examined due to the strong dimensional changes, which render them unsuitable for most applications.

**Figure 8:**
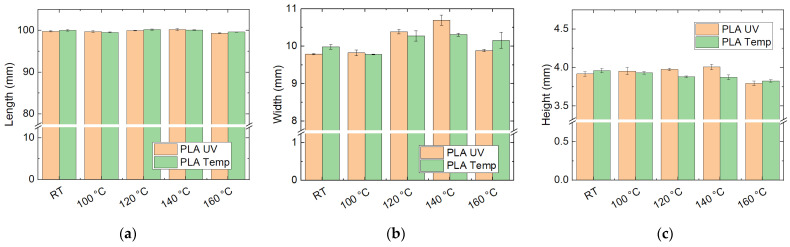
Dimensions of filled PLA samples, as printed (“RT”, room temperature) and heat-treated at different temperatures: (**a**) length; (**b**) width; (**c**) height.

Furthermore, the filled PLA filaments “UV” and “temp” were investigated after heat treatment at different temperatures. The results of the dimensional examinations are depicted in Figure 8. Here, the samples showed different behavior to the previously investigated ones. The length remained constant for both materials, i.e., no strong relaxation occurred. Similarly, the sample height was very similar to that at room temperature for heat treatment at 100 °C and even 120 °C, while a clear reduction in the height after heat treatment at 160 °C was visible. Nevertheless, no “melting” was visible even at 160 °C, in contrast to the PETG samples, although typical PLA has a glass transition temperature around 60 °C, which is far below all heat treatment temperatures used here. 

Nevertheless, the sample width changes with heat treatment. Interestingly, this effect is small at 100 °C, indicating that cold crystallization around this temperature may be a good means to prepare these materials for further thermal tests.

**Figure 9:**
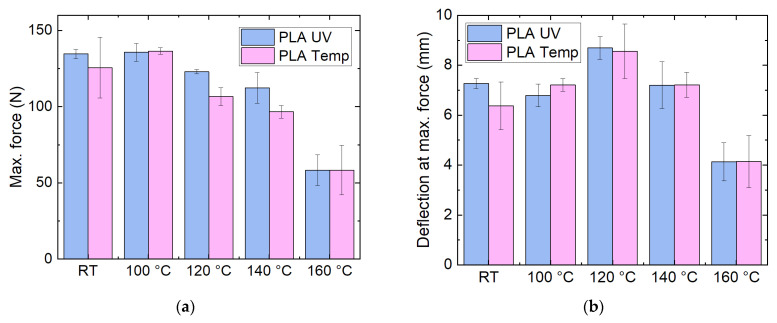
Mechanical properties of filled PLA samples, as printed (RT) and heat-treated at different temperatures: (**a**) maximum force; (**b**) deflection at maximum force in 3-point bending tests.

The results of the corresponding mechanical investigations are depicted in Figure 9. In contrast to the tests with pure PLA (Figure 5), here, no crystallization is visible; neither the maximum force nor the deflection at the maximum force show significant modifications after heat treatment. Contrastingly, the highest temperatures significantly weaken the material and should thus be avoided.

**Figure 10:**
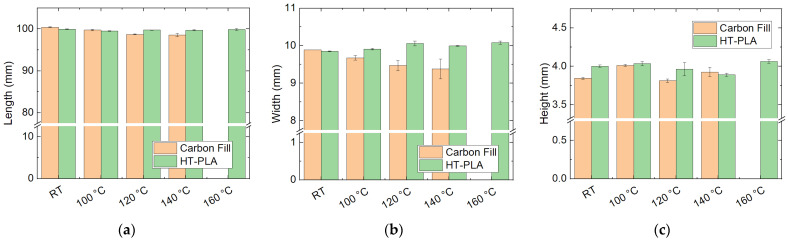
Dimensions of other filled samples, as printed (RT) and heat-treated at different temperatures: (**a**) length; (**b**) width; (**c**) height.

Moreover, the same tests were carried out with HDglass Carbon Fill—the only unmolten PETG material in the previous tests—and HT-PLA, two other promising materials. The results of the dimensional stability tests are depicted in Figure 10. In both cases, the lengths stay relatively stable for heat treatment at different temperatures, while the height generally varies for Carbon Fill, which may be attributed to the high filling of the material, sometimes resulting in deviations from the planned dimensions after printing. In addition, the width of the Carbon Fill samples is increasingly reduced with increased heat treatment temperature, including some melting at 140 °C, which is why it was not tested at 160 °C. HT-PLA, on the other hand, shows stable dimensions for all heat treatment temperatures, suggesting its potential use in high-temperature applications. 

**Figure 11:**
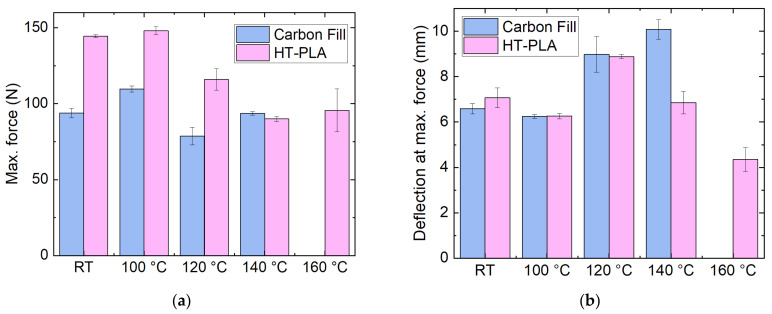
Mechanical properties of other filled samples, as printed (“RT”, room temperature) and heat-treated at different temperatures: (**a**) maximum force; (**b**) deflection at maximum force in 3-point bending tests.

The corresponding mechanical properties are depicted in Figure 11. Here, HT-PLA shows generally higher maximum forces than Carbon Fill due to the high filling of the latter. The forces remain high only, however, for heat treatment at 100 °C and are significantly reduced at higher temperatures. Similarly, the deflections at break are unaltered by heat treatment at 100 °C, while a temperature of 120 °C makes both materials more elastic, until even higher temperatures weaken the material, so that both maximum force and deflection at maximum force are reduced for HT-PLA at 160 °C. Nevertheless, for a temperature around 100 °C, HT-PLA can be expected to be as suitable as the aforementioned PLA “UV” and PLA “temp”.

### 3.3. Further Prequalification Tests with the Materials for the Final Test

**Figure 12:**
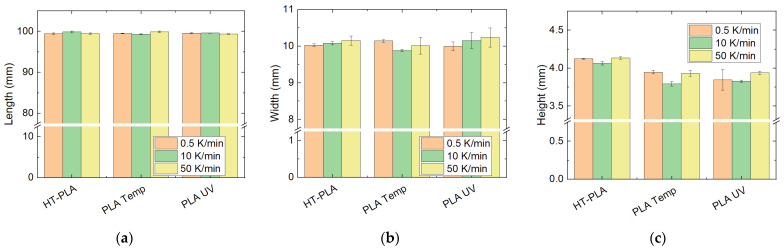
Dimensions of chosen PLA materials, heat-treated at 100 °C, approached with different heating rates: (**a**) length; (**b**) width; (**c**) height.

For the three materials chosen due to the previous results, i.e., HT-PLA, PLA “Temp” and PLA “UV”, the influence of the heating rate was additionally investigated. Figure 12 depicts the results of the dimensional tests. While the lengths are approximately identical for all chosen heating rates, the widths and heights are slightly modified with the heating rate. However, no clear trend is visible, and none of the dimensional differences for different heating rates are significant.

**Figure 13:**
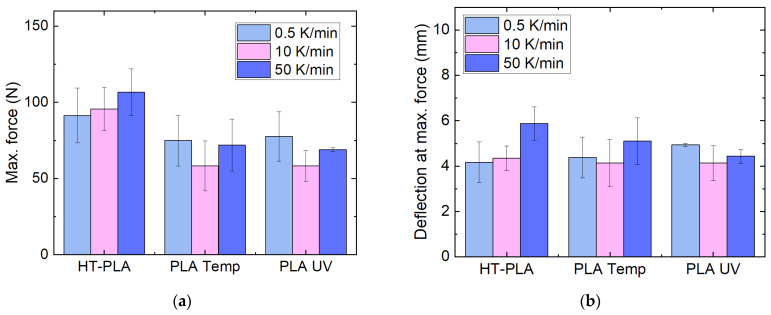
Mechanical properties of chosen PLA materials, heat-treated at 100 °C, approached with different heating rates: (**a**) maximum force; (**b**) deflection at maximum force in 3-point bending tests.

Regarding the mechanical tests, depicted in Figure 13, PLA Temp and PLA UV show slightly reduced maximum forces for the intermediate heating rate of 10 K/min. However, none of these trends is significant. For HT-PLA, on the other hand, a slight increase in the maximum force as well as the deflection at maximum force is visible. Again, this increase is not significant, as visible from the relatively large error bars.

These results indicate that the heating rate does not significantly influence the dimensional stability or the mechanical properties of the tested 3D printing polymers. All further heat treatments and DSC measurements were thus performed at the intermediate heating rate of 10 K/min.

**Figure 14:**
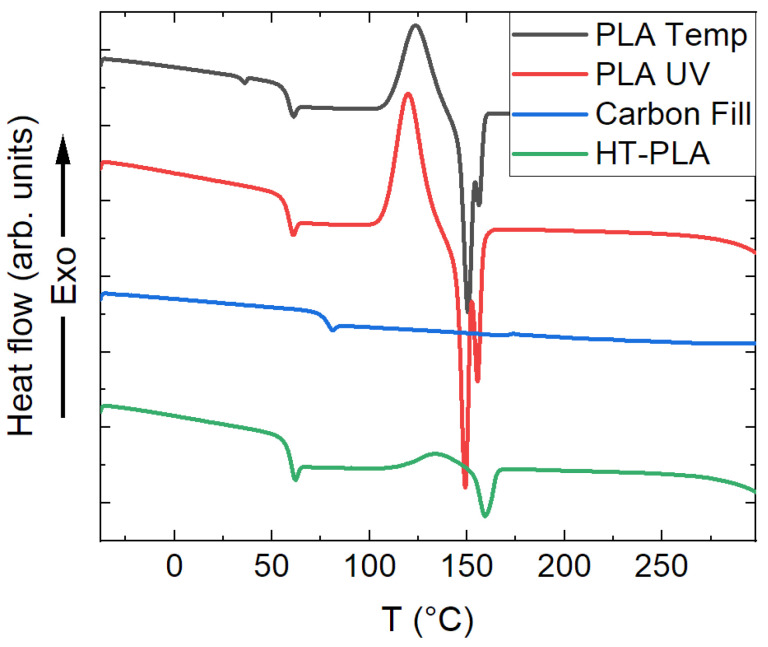
Differential scanning calorimetry (DSC) measurements of filled PLA materials.

In order to determine the temperatures that caused structural changes in the samples during repeated treatment, DSC measurements were performed on the chosen filled PLA samples, including the HDglass Carbon Fill as a reference for a sample in which no cold crystallization peak was expected (Figure 14). Indeed, all PLA materials showed a small endothermic peak with the lowered baseline around 60 °C, indicating the glass transition, while this point reached approximately 80 °C for Carbon Fill, as is typical for PETG. An exothermic peak (maximum) indicates the crystallization temperature for all PLA materials, in the case of PLA Temp and PLA UV, followed by endothermic melting peaks, which are not relevant for our study and thus not further discussed here. The crystallization maxima can be found at 127 °C (PLA Temp), 119 °C (PLA UV), and 134 °C (HT-PLA), respectively. These temperatures were chosen for the heat treatment (1 h, approached with 10 K/min) in the oven before the final tests in the climate chamber.

### 3.4. Final Tests in the Climate Chamber

**Figure 15:**
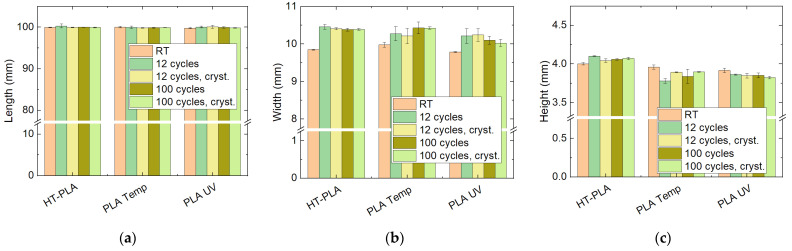
Dimensions of chosen PLA materials after cyclic thermal tests in the climate chamber: (**a**) length; (**b**) width; (**c**) height.

The dimensional changes during the final tests with HT-PLA, PLA Temp, and PLA UV in the climate chamber are depicted in Figure 15. For all materials, the lengths remain approximately identical after 12 cycles as well as after 100 cycles, both for as-printed and for post-crystallized samples. The heights are also nearly constant for HT-PLA in all cases, while they are slightly reduced for PLA Temp and PLA UV. Since PLA Temp shows the largest effect for 12 cycles without post-crystallization, it can be assumed that these changes are mostly based on arbitrary variations and not significant. Nevertheless, for PLA Temp and PLA UV, all heights after heat treatment are slightly below the heights of the as-printed samples, indicating that there is indeed a certain deformation. This is even stronger regarding the widths of the samples, where, for all three materials, the height is clearly increased upon cyclic thermal tests. For HT-PLA, however, all three dimensions are not significantly changed between 12 cycles and 100 cycles, indicating that, after a certain adaption phase, the dimensions should be stabilized. 

**Figure 16:**
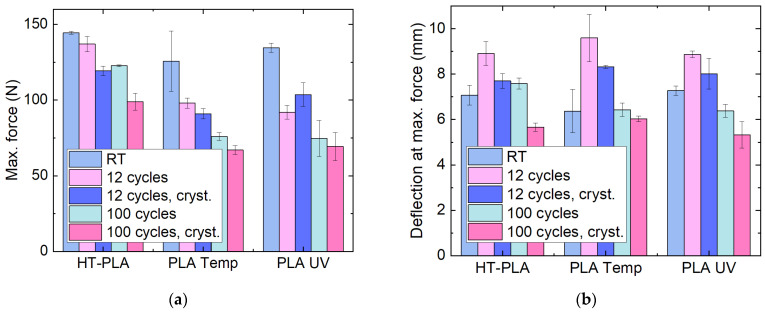
Mechanical properties of chosen PLA materials after cyclic thermal tests in the climate chamber: (**a**) maximum force; (**b**) deflection at maximum force in 3-point bending tests.

The mechanical properties, nevertheless, show a different behavior, as Figure 16 indicates. All materials show a clear reduction in the maximum force in the three-point bending tests with cyclic thermal tests (Figure 16a). The deflection at maximum force is, in all cases, largest after 12 cycles for the samples without post-crystallization. However, after 100 cycles, it decreases below the original deflection at maximum force of the as-printed samples, indicating a property deterioration with cyclic temperature changes.

**Figure 17:**
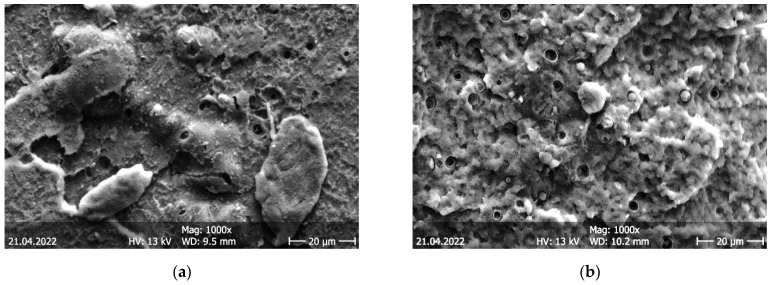
Scanning electron microscopy (SEM) images of rupture cross-sections of PLA Temp: (**a**) without thermal cycling; (**b**) after post-crystallization and 100 thermal cycles.

To understand the effect of cyclic thermal treatment on the mechanical properties, the rupture cross-sections of PLA Temp samples without thermal cycling and after post-crystallization and 100 thermal cycles are compared in Figure 17. Here, the effect of the thermal cycling is clearly visible. While both ruptures are brittle, without the typical deformation bands found after ductile fracture [32], and both images show the debonding of the microbeads responsible for the color change upon heating, the fracture surface after thermal cycling becomes rougher after 100 thermal cycles (Figure 17b), indicating the reduced toughness of the samples [33], as was found in the mechanical tests. The increased roughness in Figure 17b is visible for a scale of some micrometers, with round “hills” and “valleys”, as compared to the nearly planar layers of Figure 17a with some visible steps.

As these experiments show, HT-PLA in particular has relatively high dimensional stability upon cyclic thermal treatment. Nevertheless, the decrease in the mechanical properties prevents the use of this material or the other filled PLA materials under investigation for structural elements in applications subjected to thermal cycling. A possible solution for this problem may be forming composites of 3D-printed and embedded fibrous materials, i.e., investigating 3D- and 4D-printed polymer–textile composites [34,35] under cyclic thermal treatment.

Finally, it must be mentioned that using commercially available filaments leads to a lack of knowledge of all relevant parameters. Specifically, the molecular weight is usually not given by the producers, but it is known to have a significant effect on the mechanical properties of 3D-printed objects [36,37]. Future experiments should thus also include self-produced filaments with well-defined molecular weight. 

## 4. Conclusions

Several polymers typically used for FDM printing were investigated with respect to their dimensional stability and mechanical properties upon a single heat treatment and cyclic thermal load. Comparatively, some filled/blended PLA materials had the best dimensional stability, while most PETG materials showed melting even at relatively low temperatures, and ABS and HIPS generally had small forces at break. This indicates that even many commercially available filled/blended PLA materials can be used for autoclaving and other applications that necessitate temperatures slightly above 100 °C.

Due to the nevertheless insufficient mechanical properties after 100 cycles between −40 °C and +80 °C, these PLA materials are not appropriate for applications in LEO on their own. Hence, further research is required, suggesting the testing of composites with textile fabrics or other fibrous materials after similar thermal treatments.

## Data Availability

All data are shown in the paper.
